# Obesity in patients with acute lymphoblastic leukemia in childhood

**DOI:** 10.1186/1824-7288-38-4

**Published:** 2012-01-27

**Authors:** Lorenzo Iughetti, Patrizia Bruzzi, Barbara Predieri, Paolo Paolucci

**Affiliations:** 1 Department of Paediatrics, University of Modena & Reggio Emilia, Modena, Italy

**Keywords:** Acute lymphoblastic leukemia, children, obesity, metabolic syndrome

## Abstract

Acute lymphoblastic leukemia is the most common malignancy in childhood. Continuous progress in risk-adapted treatment for childhood acute lymphoblastic leukemia has secured 5-year event-free survival rates of approximately 80% and 8-year survival rates approaching 90%. Almost 75% of survivors, however, have a chronic health condition negatively impacting on cardiovascular morbidity and mortality. Obesity can be considered one of the most important health chronic conditions in the general population, with an increasing incidence in patients treated for childhood cancers and especially in acute lymphoblastic leukemia survivors who are, at the same time, more at risk of experiencing precocious cardiovascular and metabolic co-morbidities. The hypothalamic-pituitary axis damage secondary to cancer therapies (cranial irradiation and chemotherapy) or to primary tumor together with lifestyle modifications and genetic factors could affect long-term outcomes. Nevertheless, the etiology of obesity in acute lymphoblastic leukemia is not yet fully understood. The present review has the aim of summarizing the published data and examining the most accepted mechanisms and main predisposing factors related to weight gain in this particular population.

## Introduction

As a result of treatment, almost 4/5 of subjects with a diagnosis of cancer in childhood become long-term survivors. It has been estimated that in the United States about 1 every 640 adults between the ages of 20 and 39 years is a survivor of pediatric cancer [1]. Nevertheless, three decades after a diagnosis of cancer, almost 75% of survivors have a chronic health condition [2]. Consequently, the late side-effects of the cancer treatment have obtained an increased attention and the long-term monitoring of survivors has become an important part of their overall health care.

Obesity is a worldwide health chronic condition, affecting also cancer survivors, and is one of the most important factors increasing cardiovascular morbidity and mortality.

Acute lymphoblastic leukemia (ALL) is the most common childhood malignancy, affecting approximately 1- 4.75 per 100.000 people worldwide, with a peak age of occurrence between 2 and 6 years of age. It accounts for 80% of all leukemia cases in children [3]. Italy, United States, Switzerland and Costa Rica are the countries with the highest incidence of ALL [4]. From 1975 through 2006, ALL incidence rates increased significantly, with an annual percentage change of 0.8% [5]. Especially in children aged 1-4 years, the annual percent change reaches the 1.2% [4]. Nevertheless, recent progress in risk-adapted treatment for childhood ALL has secured 5-year event-free survival rates of approximately 80% and 8-year survival rates approaching 90% [1]. Survivors of ALL form the largest group of long-term survivors from childhood cancer, even if they have to be considered a heterogeneous group depending on the treatment. The standard treatment of childhood ALL includes different phases (induction, consolidation/intensification, and maintenance phases) in which combination chemotherapy, central nervous system (CNS) sanctuary therapy with intrathecal chemotherapy and high dose chemotherapy are given. CNS treatment has been an essential component in preventing meningeal relapse and its introduction contributed to a steady increase in long term survival [1]. In the early 1980s, the Berlin-Frankfurt-Muenster (BFM) group showed that the use of intensive induction, consolidation, and a delayed intensification phase for patients with ALL produced an approximately 70% cure rate. Subsequent trials have refined this treatment strategy, identifying the superior outcome achieved with dexamethasone versus prednisolone and identifying the superior outcomes associated with specific augmentations of the BFM regimen in post induction phase therapy. In this way, improvements in survival have been achieved while reducing the percentage of children who receive cranial radiation as a component of treatment [5]. However, also chemotherapy can lead to serious sequelae, including growth and endocrine dysfunctions and obesity.

In this review we discuss the data about obesity in survivors from ALL, considering that, due to the administration of potential cardiotoxic chemotherapy or radiotherapy, they are more prone to develop cardiovascular disease than general population.

### Obesity during ALL treatment

Children treated for ALL are normally well-nourished at diagnosis [6,7]. The first year of treatment is documented to be the period of most marked excess weight gain [8,9] and, in small cohort of patients, at the end of consolidation treatment the percentage of overweight could raised to about 50% [6]. Table 1 lists the studies analyzing the trend of weight gain during ALL treatment in childhood and adolescence. Variations between the results of the studies may be due to differences both in treatment protocols and in definitions of excess weight gain and to smallness of samples size [table 1]. In fact, many factors could influence the rate of weight gain during treatment, among which, first of all, there is the therapy itself. That explains why not all children gained excess weight on treatment. For instance, the incidence of stomatitis and gastrointestinal toxicities during consolidation and delayed intensification of increased-intensity arms (Children's Cancer Group protocol CCG 1961) may lead to weight loss [9]. Paradoxically, in the retrospective analysis conducted by Withycombe et al among 1638 patients enrolled in CCG 1961 from 1996 to 2002, no differences in weight pattern were detected in patients on an increased intensive or prolonged treatment-arm and at the end of the ALL treatment the overall percentage of overweight and obese patients increased from diagnosis [9]. Moreover, the tendency of weight gain during treatment may be influenced by weight at diagnosis. In 2005, Baillargeon described the weight gain pattern in 141 white Hispanic ALL pediatric patients during chemotherapy. For normal body mass index (BMI) sub cohort at diagnosis, BMI increased substantially between baseline and 12 months, increased moderately between 12 months and 24 months, and then showed a slight decrease at 30 months. For the ones overweight at diagnosis, BMI exhibited no consistent pattern of increase or decrease over time. For the obese sub cohort, there was described a slight overall decrease over time [10]. Gender-dependent differences in weight gain pattern have been already reported [11]. Girls become obese between diagnosis and the end of chemotherapy. Boys seem to have a progressive and gradual increase in BMI through to attainment of final height.

**Table 1:** List of studies analyzing the variation of the prevalence of obesity in childhood during ALL treatment.

Study	**Num**. Patients	Definition (BMI centile)	Obesity Prevalence (%) at Diagnosis	Obesity Prevalence (%) End of therapy
***Odame et al ***[6]	40	> 97.7th	5	43 (in girls) 26 (in boys)

***Van Dongen-Melman et al ***[7]	113	> 90th	8	30

***Reilly et al ***[77]	98	> 97.7th	2	9

***Mayer et al ***[78]	39	> 97.7th	3	38 with cranial irradiation 48 without

***Withycombl et al ***[9]	1638	> 95%	14	23

***Chow et al ***[71]	165	Overweight > 85-94% for age Obesity > 95% for age	Overweight 12.7% Obesity 10.9%	Overweight 17% Obesity 21.2%

### Obesity in ALL survivors

If the weight gain starts from the beginning of the therapy, it continues after its end [12]. Early studies by Sainsbury and colleagues suggested a clinical impression that survivors of childhood ALL tended to be overweight and obese in adulthood [13]. In a retrospective national health survey among 414 ALL survivors, a statistically significant increases in fatness occurred during the first year off therapy, at the end of which 35% of the children were above the 80th percentile of weight for age and 12% were above the 95th percentile. This distribution persisted during the subsequent 4-years follow-up period [12]. Table 2 lists the results of the studies showing the prevalence of overweight and obesity in adult survivors of childhood ALL [table 2]. However it has to keep in mind that all the published data are difficult to be interpreted due to several reasons. First of all, the analyses of auxological data in long-term childhood cancer survivors were mostly performed in cross-sectional or longitudinal studies in retrospective cohorts [14]. In addition, differences in treatment protocols, definitions of excess weight gain (use of different reference data) and the relatively small sample sizes of each study (particularly related to the heterogeneity of treatment within some studies) could influence the rate of prevalence of overweight and obesity between studies. Moreover, most studies analyzed weigh changes in children treated with both cranial irradiation and combination chemotherapy. Thus, disentangling the adverse contribution of these two major therapeutic modalities has proved difficult.

**Table 2:** List of studies analyzing the long-term prevalence of obesity in childhood after the end of ALL treatment.

Study	**Num**. Patients	Obesity definition	Assessment period	Obesity Prevalence (%)
***Schell et al ***[63]	91	BMI > 24 kg/m^2^	Final height	38

***Odame et al ***[6]	40	BMI > 97.7 th	Diagnosis + 4 ys	57 (girls) 21 (boys)

***Van Dongen-Melman ***[7]	113	BMI > 90 th	Diagnosis + 4 ys	24

***Didi et al ***[11]	114	BMI > 85 th	Final height	46

***Talvensaari et al ***[42]	50	Body weight > 120% ideal	Diagnosis + 13 ys	32 (vs.10 in controls)

***Birkebaech et al ***[34]	33	BMI > 90 th	Final height	36

***Nysom et al ***[30]	95	BMI > 90 th	Diagnosis + 11 ys	25

***Reilly et al ***[77]	98	BMI > 97.7 th	Diagnosis + 3 ys	16

***Shaw et al ***[65]	33	BMI > 85 th	Final height	56 (girls) 13 (boys)

***Sklar et al ***[28]	126	BMI ≥ 85th	Final height	Overall 30 18 Gy irradiated: 38 24 Gy irradiated: 40 Not irradiated: 30

***Mayer et al ***[78]	39 (25 irradiated; 14CT only)	NA	3.4-14.6 ys (from end of therapy)	Overall 38 (48 irradiated, 21 CT only)

***Meacham et al ***[64]	1665	BMI ≥ 30 kg/m^2^	Adulthood	18 (girls) 16.5 (boys)

***Jarfelt et al ***[31]	35	BMI 25-29.9 kg/m^2^	20 ys (minimum: 15 ys)	34

***Van Beck et al ***[50]	90	BMI ≥ 30 kg/m^2^	12.7 ys	8

***Trimis et al ***[73]	80	NA	6.3 ys	25

***Anser et al ***[79]	54 CT only	BMI > 97.7 th	Final Height	30 overweight 18 obese

***Breene et al ***[40]	77	BMI SDS > 2.3	3 ys	47.2 (vs 29.9 at diagnosis)

NA: Not Available; BMI: Body Mass Index; ys: years; CT: Chemotherapy.

### Etiology of obesity in ALL

#### Lifestyle

Environmental factors intervene in the development of obesity during and after the treatment of ALL. During treatment, patients usually undergo changes in their routinely lifestyle. Increased energy intake and reduced habitual physical activity are commonly considered the main responsible factors of weight gain. The loss of physical activity may start during the hospitalization of the patient. It may be due to a number of factors, including diminished exercise capacity, impaired motor function, diminished interest in recreational activity and over-protectiveness of the child's primary caregivers. Pathophysiological changes in the cardiorespiratory system [15] or growth hormone insufficiency might also contribute to reduce physical activity as well as the presence of steroid-related myopathy and vincristine-related neuropathy. In a study of Reilly and colleagues in 1998, the total energy-expenditure (TEE) was measured using doubly-labeled water and resting energy-expenditure (REE) by indirect calorimetry. Energy expended on physical activity was calculated as TEE:REE. They found a higher TEE in controls than in 20 ALL patients treated according to UK ALL XI protocol. In the same way, TEE:REE was demonstrated higher in controls than in patients [16]. After the end of the ALL treatment, the persistence of an imbalance between energy intake and physical activity plays a central role in determining or carrying on obesity. Warner et al reported differences in TEE, evaluated by monitoring heart rate, REE and energy expended on physical activity in 31 survivors of ALL, 21 survivors of other childhood malignancies and 32 healthy sibling controls. The engagement in physical activity was reduced in ALL survivors in comparison with both healthy siblings and survivors other malignancies [17]. The author suggested that the decreased in TEE could derive from the long-term cardiac damage of anthracyclines. Marinovic et al studied physical activity in 37 ALL survivors (all treated with prednisone, but without cranial irradiation) at 2.2 and 3.2 years post-treatment. At the first evaluation, survivors were less physical active and had a higher median body fat compared to controls, while mean BMI was only slightly higher. At the last follow-up, physical activity and body fat were similar in ALL-survivors and matched controls [18]. Caloric intake, physical activity and changes in REE during and after therapy could be influenced by the modalities of treatment received. In regards to energy intake, there are only limited evidences on its increasing in patients during treatment for ALL [19-21]. Nevertheless, it has been already documented that dexamethasone therapy can increase energy intake and decrease physical activity [22]. The finding supports the hypothesis that hyperphagia, induced by hypothalamic insult or corticosteroid effects, can be an important contributor to obesity in ALL.

#### Hypothalamic damage radiotherapy-induced

Hypothalamus damage cranial irradiation-induced might be involved in weight gain in ALL. Radiotherapy can damage hypothalamus leading to hormonal deficiency and hypothalamic dysregulation of food intake control. The effect of radiotherapy on weight gain in childhood ALL survivors was studied in small cohorts, with conflicting data. In the study of Craig and colleagues, a severe increased BMI z score at final height was documented in female survivors treated with lower dose (18-20 Gy) of cranial irradiation: 14% of these patients had a BMI greater than 30 kg/m^2 ^in their early adult life [23]. The finding suggested a dose- and gender-dependent deleterious effect of cranial irradiation on CNS function. This finding was confirmed later by other authors [24]. The low dose of irradiation could damage the more radiosensitive pathway of CNS, in which hypothalamus is included, involving the regulation of thermogenesis or satiety. Moreover, the gender-dependence of effects of irradiation could be related to different age of cerebral development and vulnerability of the nervous structure in boys and girls. According to this, irradiated ALL survivor girls seemed to be particularly affected by precocious puberty and to have experienced greater growth impairment than boys [25,26]. The study conducted by Oeffinger and colleagues achieved similar results, even if they detected an opposite and direct dose-dependent radiotherapy-effect. In fact, the authors demonstrated that the age- and race adjusted odds ratio (OR) for obesity in survivors treated with cranial radiation therapy ≥ 20 Gy in comparison with siblings was 2.59 and 1.86 for females and males, respectively. Furthermore, they underlined that overweight or obesity was not associated with treatment consisting of chemotherapy only or radiation doses of less than 20 Gy [27]. To confirm the data, Sklar et al found a greater increase in BMI in survivors treated with 24 Gy radiation compared with those treated with 18 Gy [28].

Some authors consider BMI not the best indicator of obesity in patients with chronic diseases. Therefore, in some studies, the evaluation of fat adiposity was determined by other indicators, including body %fat, measured at DEXA, skinfold thickness and waist- and hip-circumferences. Even if some results of DEXA-studies show higher %fat in survivors treated with cranial irradiation [17,29], data are still conflicting. In 1999, data from 95 ALL survivors followed for about 11 years after the diagnosis were published [30]. Even if at the end of follow up, BMI did not differ significantly between patients and 463 controls, a %fat above the 90^th ^percentile of the reference value was found through DEXA in 26% of ALL survivors. Adjusted for sex and age, a higher %fat was related to cranial irradiation and GHD, but not to gender, the cumulative dose of antrhacyclines or corticosteroids or the type of corticosteroids used [30]. Warner et al measured skinfold thickness together with BMI and %fat mass at DEXA in 35 ALL-survivors treated with cranial irradiation. Skinfold thickness as well as whole body %fat was increased in 21 females, but not in males, after an average of 7 years after ALL treatment [17]. Jarfelt et al measured waist- and hip-circumferences in ALL-survivors. Besides an increased BMI and an increased %fat mass at DEXA, they also found significantly higher waist circumference and higher waist-to-hip ratio in radiotherapy-treated men compared with men treated without radiotherapy [31]. In childhood, fat-free mass (FFM) is different from the one in adulthood because it has a lower density and a greater hydration. Therefore, in a cross-sectional study conducted by Murphy and colleagues, FFM of 24 children in remission from ALL were evaluated by using the 4-component model technique (anthropometry, air-displacement plethysmography, deuterium dilution and DEXA) [32]. The study showed that, at an average of 4 years after the diagnosis of ALL, patients had a marked increased BMI, FFM, fat mass, %fat mass and fat mass index in comparison with age-matched, healthy controls.

#### Chemotherapy

Even if there are evidences that the risk of obesity is higher for patients treated on older protocols which included cranial irradiation, also children treated with more modern protocols experienced weight gain. Patients not irradiated could be considered a tool to distinguish the effects caused by radiotherapy from those caused by chemotherapy. Vaisman showed already in 1993 that specific form of chemotherapy, like methotrexate and 6-mercaptopurine, affects metabolic fuel utilization and protein synthesis and turnover, altering body composition [33]. In 1998 Birkebaek and Clausen observed weight gain during the ALL treatment period indicating that chemotherapy could be a major factor in the etiology of weight gain during treatment [34]. In contrast, before the end of the treatment, some evidences of recovery of BMI SDS to baseline levels were documented [35]. In 2003 Oeffinger and colleagues published data documenting that no chemotherapeutic agent, either individually or in combination, was significantly associated with overweight and obesity in ALL survivors during and after treatment [27]. Long-term BMI data in an unirradiated population are not yet fully described and, as well as short-term data, they are extrapolated from small population. In 1995, Ventham and colleagues examined changes in BMI SDS up to 6 years after diagnosis in 126 ALL patients diagnosed between 1990 and 1997. They were not treated with cranial irradiation (UK ALL XI protocol). There was a significant increase in BMI SDS from diagnosis to 3 years after it in 59 ALL patients and the prevalence of obesity increased from 1.7% at diagnosis to 15.3% at 3 years after diagnosis [36]. In the study of Craig and colleagues unirradiated patients had raised BMI Z score at the latest follow-up (more than 4 years after the end of treatment) [23]. The rate of weight gain varies between studies: Garmey and colleagues reported a percentage of obesity, after few years from chemotherapy for ALL, varying between the 15% and 22% and the 9.1% and 19.9% in males and females, respectively [37]. Razzouk in 2007 documented that 33.7% of 101 young adults treated previously with chemotherapy-alone for ALL were obese [38]. In 2003 Dalton and colleagues evaluated the long-term (median follow-up time from diagnosis of ALL 6.1 years) weight changes in 618 ALL children treated with different CNS therapies: intrathecal therapy alone, intrathecal therapy with conventional cranial irradiation or intrathecal therapy with twice-daily radiation. Children younger than 13 years at diagnosis had a statistically significant increase in their BMI z scores, regardless of CNS therapy they received [39]. The same results were achieved by Van Dolgen-Melman [7] and Razzouk and colleagues [38]. The first group documented that BMI increased both in patients treated with cranial irradiation and in patients treated without irradiation but with higher intracranial methotrexate doses [7]. Razzouk, reporting the results of a retrospective longitudinal study performed in 456 children with a new diagnosis of ALL or lymphoma and enrolled into a single protocol, did not find a significant difference in BMI between patients who received radiation and those who did not [38]. Recently, Murray and his group report data from a retrospective long-term follow-up of 77 patients with a diagnosis of ALL at a median age of 4.6 years, treated on UKALL XI protocol (without cranial irradiation as standard therapy). Recording height and weight of each enrolled patient annually from diagnosis of ALL until 3 years after the end of chemotherapy, they documented that in the whole group BMI-SDS increased from the beginning of the treatment and was still raised at the time of last visit with a difference between genders: in males, BMI-SDS was transient, recovering by the second years after the end of chemotherapy, whereas in females BMI increases did not appear until the end of the treatment, but then it persisted until the last follow-up visit [40]. In the unirradiated cohort from Cambridge, the frequency of overweight and obesity, as determined by world health organization (WHO) standards, approaches 50% 3 years after the end of chemotherapy (*vs*. 30% at diagnosis)[40].

#### Growth hormone deficiency

GH deficiency (GHD) is the most frequent endocrine dysfunction observed following cranial radiotherapy and its metabolic effects include metabolic syndrome-like disorders, such as visceral obesity, hypertriglyceridemia, low high-density lipoprotein (HDL)-cholesterol, coagulopathy and hypertension [41]. Whether GHD contributes to obesity in ALL survivors is controversial. Considering that the major increase in BMI occurred at a time (on treatment) when most irradiated individuals are still able to produce normal amounts of GH, it is unlikely that radiation-induced GHD is responsible of early changes in body composition. However, it seems plausible that growth hormone deficiency might play a role in the maintenance of increased adiposity once it is established [28]. In an analysis of 50 childhood cancer survivors, including 28 ALL survivors, reduced spontaneous GH secretion was associated with obesity [42]. A negative correlation between trunk %fat and physiological GH secretion was also documented in 19 patients, aged 22-32 years and treated with cranial radiotherapy for ALL in childhood [31]. In contrast, Adan et al did not find a significant association between obesity and GHD in an analysis of 90 young adult cancer survivors (28 ALL survivors) [24]. GHD could occur also in unirradiated patients and contributes in weight gain. Birkebaek et al found GHD in 2 out of 11 ALL-survivors treated with chemotherapy only [43]. The data were confirmed by Gurney et al showing the about 20% of ALL adult survivors treated without any cranial irradiation-treatment was affected by GHD [44]. Thus, the presence of a central effect of chemotherapy alone seems to be confirmed and it can act through impairment of GH secretion and lead to adverse metabolic consequences in young adult survivors.

#### Corticosteroids

Of the drugs used to treat ALL, glucocorticosteroids are the only one with a known effect on weight gain. Corticosteroids can promote obesity via a range of possible mechanisms: effects on appetite/regulation of energy intake; alteration in substrate oxidation and alterations in energy expenditure. Other theories are that glucocorticoid treatment causes an increased adiposity by suppressing growth hormone secretion [45] or that it causes resistance to leptin [46]. Even if the role of corticosteroids was suspected long time ago, only recently some empirical evidences of energy balance effects of corticosteroid therapy have been demonstrated. Nevertheless, the results of the studies reported in literature have to be interpreted in relation with the changes in corticosteroid dosing between the treatment eras. In fact, since the 1970s, cumulative corticosteroid dosing has increased by as much as 60% to 80% with the addition of corticosteroid pulses during the postinduction delayed intensification treatment phase and during the maintenance phase for a larger number of patients. In addition, over the past 15 years the use of dexamethasone in ALL protocols has been increased. Although the per-dose glucocorticoid equivalency of dexamethasone is similar to prednisone, dexamethasone differs from prednisone in other aspects, including a substantially longer duration of action and improved CNS penetration, as well as a higher incidence of avascular necrosis, hyperglycemia and myopathy [47]. Therefore, suspecting the presence of more side-effects in dexamethasone protocols, various attempts have been made to relate degree of weight gain to the dose or type of steroid used. These were largely inconclusive, usually because of relatively small sample sizes. In a study from UK conducted by Reilly et al, the energy intake in children treated for ALL was significantly increased during the maintenance phase. The magnitude of the increase in energy intake was substantial, around 20% on average, during 5-day course of dexamethasone (6.5 mg/m^2^) or prednisolone (40 mg/m^2^). Even if a trend for a more marked increase in energy intake during dexamethasone therapy compared to prednisolone was detected, no statistical significance was demonstrated [48]. Considering these results, it is logical to suspect that, during the intensification phase, when glucocorticoids are used for a longer period than in maintenance, the contribution to positive energy balance may be even greater. van Dongen-Melman demonstrated, by comparing BMI SDS up to 4 years after treatment for ALL in 6 treatment protocols, that ALL protocols with the highest glucocorticoid dose induced the highest weight gain. Nevertheless, the effect of dexamethasone on increasing BMI seemed to be temporary [7]. Van der Sluis et al. found no abnormal body composition at DEXA after mean follow-up of 9.6 years post-treatment, despite high-dose dexamethasone during therapy [49]. Also Nysom et al [30] and Jarfelt et al [31] found no increase in fat mass after corticosteroid treatment at an average of 7.6 years and 20 years, respectively. Van Beek et al showed that fat was increased in prednisolone-treated survivors 12.7 years post-diagnosis, whereas it was normal in the dexamethasone-treated group [50]. Opposite results were found by Groot-Loonen and colleagues [51]. In Dalton's study, the post-induction corticosteroid treatment varied between groups (prednisolone *vs*. dexamethasone). However, there was no statistically significant difference in patterns of obesity between the groups [39]. No statistical difference in fat mass, fat-free mass and their index between dexamethasone and prednisolone groups was found after the end of ALL treatment by Murphy and colleagues using a 4-component reference model [32]. Individual and genetic factors may affect the effects of glucocorticoids on weight. In fact, a selective polymorphism in the glucocorticoid receptor gene may increase the sensitivity to glucocorticoid effects, including weight gain [9].

#### Premature adiposity rebound

One mechanism that might contribute to the long-term obesity in survivors of ALL is early adiposity rebound (AR). The AR is the period of childhood (typically between 5 and 7 years) when BMI and other indices of adiposity begin to increase after reaching their nadir [52]. The earlier the age at which AR occurs, the more likely a child is to be overweight or obese as an adult [53]. Although the mechanisms that underlie this process are unknown, it is clear that the AR is a critical period for the development of adult obesity [54]. Because peak incidence of ALL occurs around the time of the AR and excess weight gain is typical in ALL patients, particularly in the first year of treatment, it seemed plausible that patients with ALL would be characterized by premature AR. In literature, only one study investigated the timing of AR in 68 patients with ALL treated before the age of 30 months [55]. In this study, respectively, 42.6% and 80.9% of the survivors showed AR at the age of 3 and 4 years, while, respectively, 4.5% and 21.2% happened in controls (p < 0.001). It demonstrated that children with ALL can experience AR much earlier than their healthy peers. The influence of AR on the future risk of becoming obese is related to its timing, but not with the BMI when AR occurs. Therefore, the positive energy balance characteristic of treatment for ALL is sufficient to produce early AR, even in children not obese or overweight, and it could affect weight later in life.

#### Leptin

Leptin, an adipocyte-derived hormone, is the product of the *ob *gene and regulates appetite and energy expenditure. It is produced in adipose tissue, released into the circulation and binding specific hypothalamic receptors, it acts as a hormonal feedback signal to regulate food intake and metabolic rate. Normally, an increase in leptin level causes a decreased appetite and food intake and increased energy expenditure. With increasing fat mass, leptin level increases exponentially, thus reflecting the amount of stored fat. Above a threshold of 25-30 ng/ml, serum leptin levels are not translated into proportional increases in cerebrospinal or brain leptin levels. This, in turn, may result in leptin resistance and obesity. Most obese have increased leptin levels, indicating that in most of them obesity is a leptin-resistant state [56]. Probably a disruption of the normal relationship between leptin and BMI exists also in ALL, as confirmed by Davies et al [46]. In the study of Arguelles and colleagues, in which 26 prepubertal children with ALL were studied for 36 months after diagnosis, a significant positive correlation was found between serum leptin levels and every anthropometric parameter monitored during the follow up (weight, BMI, skinfolds). Serum leptin levels were found elevated 6 months after diagnosis and 1 year after chemotherapy withdrawal. The first rise in serum leptin levels seemed to be secondary to the rise in body fat and the use of corticosteroids, which stimulate leptin synthesis [8]. Wallace et al performed a longitudinal prospective study of circulating leptin levels and BMI in 19 ALL-children during the first 16 weeks of treatment. A narrow correlation was found between serum leptin concentrations and BMI, and after 4 weeks of high dose steroids the leptin/BMI ratio was increased [57]. Arguelles and colleagues found no differences in serum leptin levels between girls and boys and between irradiated and unirradiated patients. By contrast, in Birkebaek's study, serum leptin was significantly higher in patients treated with cranial irradiation compared with the non-irradiated group [43]. These data confirmed the results of the study of Brennan et al [58] and the recent studies of Skoczen [59] and Karaman and colleagues [60]. The latter, studying 93 survivors of childhood ALL after a follow-up of 10.21 ± 4.90 years, demonstrated a significantly increased BMI only in the group treated with cranial irradiation and higher leptin levels only in females belonging to this group than control females. With the purpose of explaining these data, it has been already speculated that radiation-induced damage to the pituitary-hypothalamus axis may result in a disruption of leptin signal. Moreover, a sex-related difference in leptin concentration was already known in literature [29,61]. Leptin receptor polymorphism (*LEPR Gln Arg polymorphism*) may influence the response to the exposition to cranial radiotherapy and, thus, the individual susceptibility to obesity. The data from Ross and colleagues support the hypothesis that female ALL survivors who are homozygous for the Arg genotype of leptin receptor gene and treated with cranial radiotherapy have lower leptin binding affinity, higher levels of leptin and, consequently, a higher risk of become obese [62].

#### Other Risk Factors

A part from the variety of elements already taken into consideration, others can be implicated in determining excess body fat in survivors of ALL. In the study of Reilly and Ventham in 1998, the authors identified low BMI SDS at diagnosis, younger age at diagnosis and female gender as risk factors for later excess weight gain [36]. Despite reaching statistical significance, the strength of these associations did not allow the authors targeting only those patients at high risk of obesity and they concluded that all patients with ALL should be considered at risk of becoming obese. In fact, following studies only partially confirmed these previous findings. Weight SDS greater than 0 and greater than height SDS at 1 year after the end of chemotherapy was documented to be a risk factor for obesity at 18 years of age [63]. BMI weight category at diagnosis, rather than type of CNS treatment received, was confirmed being a predictor of adult weight in the recent Razzouk's study [38]. Dalton demonstrated that the chemotherapy intensity and younger age at diagnosis had to be considered risks for weight gain [39]. The presentation of grade 3 or 4 pancreatitis/glucose toxicities during induction, interpreted as markers of steroid sensibility, were identified by Whitycombe among the risk factors of being obese, together with a high BMI at diagnosis [9]. In the large database of the Childhood Cancer Survivor Study, analyzed by Meacham and colleagues, the subjects with ALL at highest risk for obesity were female patients diagnosed at ≤ 4 years of age and who received ≥ 20 Gy of cranial radiation [64]. Other authors confirmed that in females, the prevalence of obesity and mean BMI were inversely influenced by age at diagnosis [27]. The older a patient was at diagnosis, the less likely she would be obese in adulthood. This association was not confirmed in males [27]. Garmey and colleagues confirmed that the group with the greatest increase in BMI after ALL treatment was women treated with cranial radiotherapy before the age of 10 years [37].

Genetic and social influences have not to be forgotten. Withycombe individualized black or Hispanic race as the more at risk of developing obesity throughout treatment [9]. Moreover, a maternal predisposition to obesity was demonstrated being a risk factor for the development of obesity. Among 14 obese female ALL survivors, 59% had obese mother and 14% had obese fathers [65].

#### Persistence of overweight/obesity and metabolic consequences

The excess weight gain observed in children treated for ALL persists and 40-50% of young adult survivors are still obese [Table 2]. In literature, the evidence that central body fat distribution is associated with cardiovascular risk factors is already well demonstrated [66]. Central obesity is considered one of the constellations of disorders defining the metabolic syndrome (MS) together with insulin resistance, elevated plasma glucose, dyslipidemia, hypertension and a prothrombotic and proinflammatory state. MS is an important risk factor for cardiovascular disease. According to a statement developed by the American Heart Association along with the Council for Cardiovascular Disease in the Young, based on the study of several pediatric states and their risk for cardiovascular disease (CVD), post-cancer treatment survivors were classified as tier III, which means with an increased cardiovascular risk factor, with epidemiological evidence for manifest CVD early in adult life, but after 30 years of age [67]. Among survivors of childhood ALL treated before 1990, approximately 20% had increased blood pressure and/or markers of insulin resistance, whereas the prevalence of obesity and dyslipidemia was approximately 30% [68]. The prevalence of metabolic syndrome was 16% among adolescent and young adult Finnish survivors of pediatric cancer (56% of whom had ALL) compared with 0% among population controls [42]. A part from the role of obesity, in survivor population also various hormones deficiencies, changes of insulin sensitivity, lipid metabolism, inflammatory mediators and adipokines, as well as reduced physical activity, may be altered by tumors themselves or by various cancer therapies and may lead to MS [69]. In fact, systemic treatment, like chemotherapy, may contribute to MS in several ways: damage of endocrine organs or non-hormonal system, such as magnesium metabolism, as well as endothelial and adipose tissue dysfunction [67]. In 2005 Kourti and colleagues investigated 52 survivors from ALL (median age 15.2 years, 37 months later the completion of therapy) treated according to the ALL-BFM chemotherapy without receiving cranial radiotherapy. Three criteria for the metabolic syndrome (high triglyceride levels, glucose intolerance, and obesity) were fulfilled by only three subjects (5.76%), even if twenty-nine subjects (55.7%) had at least one risk factor for metabolic syndrome [70]. In addition, increased corticosteroid exposure was associated with both increased BMI and blood pressure in Chow's study [71]. In fact, corticosteroid can promote inotropic and vasocostrictive effects on the cardiovascular system. Radiotherapy can increase the predisposition to CVD due to the potential consequent growth hormone deficit [72,73]. Gurney and colleagues, following up 75 long-term childhood ALL survivors from the Childhood Cancer Survivor Study (CCSS), demonstrated that 60% of subjects treated with cranial irradiation, compared with 20% of those who were not, had presented 2 or more components of MS and that untreated abnormally low GH levels were present in 85% of irradiated patients [44]. Similarly, Link et al evaluated GH deficiency and cardiovascular risk factors in 44 adult survivors of childhood ALL, all of whom were treated with cranial irradiation. All the patients were either GH-deficient or GH-insufficient and had a significant greater occurrence of dyslipidemia, insulin resistance, increased fat mass and a marked reduction in cardiac dimensions and performance [29].

A part from the related metabolic consequences, obesity can affect outcomes in ALL. In fact, obese patients are considered at higher risk of relapse in comparison than normal weight patients, especially if they are older than 10 years of age at diagnosis [74]. In pediatric acute myeloblastic leukemia, obese patients have greater treatment-related mortality and inferior survival compared with patients not obese [75]. It may be related to an inverse relationship between weight and plasma drug levels or to the effects on anticancer activity and toxicity of chemotherapy of several growth factors and lymphokines, all secreted by adipocytes.

## Conclusion

All these findings call for measures to prevent obesity. Considering that lifestyle is the unique modifiable factor so far, the promotion of a healthy lifestyle among survivors is imperative. Moreover, the guidelines on diet, nutrition and cancer prevention developed by the American Cancer Society are a useful tool for the maintenance of health [76]. However, it is important to keep in mind that since the diagnosis of ALL a special attention has to develop on metabolic and nutritional findings of the leukemic patient.

## Abbreviations

ACTH: Adrenocorticotropic Hormone; ALL: Acute Lymphoblastic Leukemia; AR: Adiposity Rebound; BFM: Berlin-Frankfurt-Muenster group; BMI: Body Mass Index; CCG: Children's Cancer Group; CSN: Central Nervous System; CVD: Cardiovascular Disease; DEXA: Dual Energy X-Ray Absorptiometry; FFM: Fat Free Mass; GH: Growth Hormone; GHD: Growth Hormone Deficiency; HDL: High Density Lipoprotein; MS: Metabolic Syndrome; OR: Odds Ratio; REE: Resting Energy-Expenditure; SDS: Standard Deviation Score; TEE: Total Energy-Expenditure; TSH: Thyroid Stimulating Hormone; WHO: World Health Organization.

## Competing interests

The authors declare that they have no competing interests.

## Authors' contributions

All the authors contributed to the conception of the review and were involved in writing, revising and approving the final draft of the manuscript.
